# Mobile App Intervention of a Randomized Controlled Trial for Patients With Obesity and Those Who Are Overweight in General Practice: User Engagement Analysis Quantitative Study

**DOI:** 10.2196/45942

**Published:** 2024-02-09

**Authors:** Vera Helen Buss, Margo Barr, Sharon M Parker, Alamgir Kabir, Annie Y S Lau, Siaw-Teng Liaw, Nigel Stocks, Mark F Harris

**Affiliations:** 1 Centre for Primary Health Care and Equity University of New South Wales Sydney Australia; 2 Australian Institute of Health Innovation Macquarie University Sydney Australia; 3 School of Population Health University of New South Wales Sydney Australia; 4 Adelaide Medical School University of Adelaide Adelaide Australia

**Keywords:** health literacy, primary health care, mobile application, overweight, vulnerable populations, health behavior, mHealth, obesity, weight loss, mysnapp app, mobile phone

## Abstract

**Background:**

The Health eLiteracy for Prevention in General Practice trial is a primary health care–based behavior change intervention for weight loss in Australians who are overweight and those with obesity from lower socioeconomic areas. Individuals from these areas are known to have low levels of health literacy and are particularly at risk for chronic conditions, including diabetes and cardiovascular disease. The intervention comprised health check visits with a practice nurse, a purpose-built patient-facing mobile app (mysnapp), and a referral to telephone coaching.

**Objective:**

This study aimed to assess *mysnapp* app use, its user profiles, the duration and frequency of use within the Health eLiteracy for Prevention in General Practice trial, its association with other intervention components, and its association with study outcomes (health literacy and diet) to determine whether they have significantly improved at 6 months.

**Methods:**

In 2018, a total of 22 general practices from 2 Australian states were recruited and randomized by cluster to the intervention or usual care. Patients who met the main eligibility criteria (ie, BMI>28 in the previous 12 months and aged 40-74 years) were identified through the clinical software. The practice staff then provided the patients with details about this study. The intervention consisted of a health check with a practice nurse and a lifestyle app, a telephone coaching program, or both depending on the participants’ choice. Data were collected directly through the app and combined with data from the 6-week health check with the practice nurses, the telephone coaching, and the participants’ questionnaires at baseline and 6-month follow-up. The analyses comprised descriptive and inferential statistics.

**Results:**

Of the 120 participants who received the intervention, 62 (52%) chose to use the app. The app and nonapp user groups did not differ significantly in demographics or prior recent hospital admissions. The median time between first and last app use was 52 (IQR 4-95) days, with a median of 5 (IQR 2-10) active days. App users were significantly more likely to attend the 6-week health check (2-sided Fisher exact test; *P<.*001) and participate in the telephone coaching (2-sided Fisher exact test; *P=.*007) than nonapp users. There was no association between app use and study outcomes shown to have significantly improved (health literacy and diet) at 6 months.

**Conclusions:**

Recruitment and engagement were difficult for this study in disadvantaged populations with low health literacy. However, app users were more likely to attend the 6-week health check and participate in telephone coaching, suggesting that participants who opted for several intervention components felt more committed to this study.

**Trial Registration:**

Australian New Zealand Clinical Trials Registry ACTRN12617001508369; https://www.anzctr.org.au/Trial/Registration/TrialReview.aspx?id=373505

**International Registered Report Identifier (IRRID):**

RR2-10.1136/bmjopen-2018-023239

## Introduction

### Problem Statement

Obesity is a major contributor to disease burden, increasing the risk of chronic conditions, including ischemic heart disease, stroke, diabetes mellitus, chronic kidney disease, and hypertensive heart disease [1]. According to the Global Burden of Disease Study 2017 [2], high BMI was the cause of 4.72 million deaths and 148 million disability-adjusted life-years worldwide, making it the fourth leading risk for mortality in 2017. In 2017 to 2018, an estimated 36% of the Australian adult population were overweight (ie, BMI 25.0-29.9) and 31% of them had obesity (ie, BMI≥30.0) [3]. The proportion of people who are overweight or hose with obesity is higher in populations from lower socioeconomic backgrounds [3]. In 2017-2018, 72% of Australian adults residing in the lowest socioeconomic areas were overweight or had obesity compared to 62% from the highest, after adjusting for age [3]. People from the lowest socioeconomic areas were 1.9 times more likely to have diabetes in 2020 and 1.6 times more likely to have self-reported coronary heart disease in 2017-2018 than those from the highest socioeconomic areas [4].

### Rationale for the Study

Other research has shown that mobile app-based interventions can facilitate weight loss in individuals who are overweight and those with obesity, but it requires regular app use. For example, Patel et al [5] reported that consistent weight self-monitoring via a mobile app could lead to clinically meaningful weight loss. However, the study classified only a quarter of participants as consistent trackers, which they defined as self-monitoring weight and diet on at least 6 days per week for at least 75% of the study weeks [5]. Their study highlighted that consistent tracking was crucial, but only a minority of participants did so. Similarly, Laing et al [6] found that providing access to a weight loss app to primary care patients who are overweight and those with obesity did not lead to significant weight loss compared to usual care. Only one-third of them logged into the app in the sixth month of the intervention, in which the median number of logins was 0 (IQR 0-2). The authors concluded that prescribing self-monitoring apps for caloric counting may be successful in primary care patients who are particularly motivated to lose weight [6]. Chin et al [7] analyzed user data from a popular commercial weight loss app and found that in a multivariate logistic regression model, the frequency of entering body weight and consumption of dinner particularly was associated with successful weight loss in app users. Considering other studies, the focus of this study was understanding how participants used a mobile app within the Health eLiteracy for Prevention in General Practice (HeLP-GP) trial and if its use led to improvements in health literacy and diet.

### Description of the Intervention

The HeLP-GP trial was a behavior change intervention developed for implementation in Australian general practices aimed at Australians who are overweight and those with obesity from lower socioeconomic areas to help them reduce their weight. The intervention was based on the 5As framework (assess, advise, agree, assist, and arrange) [8]. It included health check visits with a practice nurse based on the 5As framework, the use of a purpose-built patient-facing mobile app called *mysnapp*, and referral to health coaching via the “Get Healthy” information and coaching service [9]. The *mysnapp* is based on a web-based platform developed by Lau et al [10].

The trial was a pragmatic, 2-arm, unblinded cluster randomized controlled trial, which continued for 12 months. Primary outcomes included changes in weight, blood pressure, health literacy, and eHealth literacy [11,12]. Secondary outcomes included lipids, diet (fruit and vegetable intake), level of physical activity, quality of life, advice received, and referral for diet, physical activity, and weight loss [12]. Participants who received the intervention could choose to use the mobile app and access the telephone coaching program. The HeLP-GP trial assessed the intervention’s effectiveness [12]. The intervention led to significant improvements at 6 months compared to the controls for health literacy (mean DiD 0.22, 95% CI 0.01-0.44) and diet (mean DiD 0.98, 95% CI 0.50-1.47). There were no associations with any of the other outcomes [12].

### Objectives

The overall aim of this study, within the HeLP-GP trial, was to assess *mysnapp* app use, engagement, its association with other intervention components, and its association with study outcomes shown to have significantly improved (health literacy and diet) at 6 months.

Our objectives were to (1) explore differences in demographics and hospital admissions between participants who used the app and those who did not, (2) examine the duration and frequency of app use (app engagement) by participants overall and by module, (3) assess the association among app use, app engagement, and participation in other intervention components, and (4) examine the association between app use and app engagement on study outcomes that were shown to be significantly improved at 6 months (ie, health literacy and diet).

## Methods

### Ethical Considerations

The University of New South Wales Human Research Ethics Committee (HC17474) approved the trial. The University of Adelaide Human Research Ethics Committee ratified this approval. All participants provided consent to take part in this study.

### Intervention

The methodology of the randomized controlled trial, of which this study is a subanalysis, was published previously [13] and prospectively registered with the Australian New Zealand Clinical Trials Registry (ACTRN12617001508369). In 2018, a total of 22 general practices were recruited from 2 Australian states, New South Wales (South West and Central Sydney) and South Australia (Adelaide), and randomized by cluster to the HeLP-GP intervention (11 practices) or usual care (11 practices). General practices were recruited through the local Primary Health Networks. Practices were located in local government areas with Socio-Economic Indexes for Areas scores [14] equal to or below the eighth decile. The Australian Bureau of Statistics reported that these are usually associated with lower health literacy levels in the population [15], with health literacy being defined by the Australian Institute of Health and Welfare in their latest Health Literacy Report as “how people access, understand and use health information in ways that benefit their health” [16]. In total, 4 strata based on the practice size (<5 general practice [GPs] and ≥5 GPs) and the state were created and then we randomly allocated practices to each stratum’s intervention or usual care group. The intervention comprised a practice nurse–led health check; additionally, participants could choose whether to take up a lifestyle app, a telephone coaching program, or both. Potential participants were identified using the GPs’ software. The general practitioners of the intervention sites also assessed their patients for eligibility. The eligible patients were provided with trial information and consent forms by the reception staff. Recruitment occurred between October 2018 and September 2019.

At the baseline health check, the practice nurses helped participants with the *mysnapp* setup and access the coaching program. They entered the participant’s height, weight, waist circumference, and blood pressure into the app and set the health goals with the participant. For 6 weeks, the participants received a nutrition-related and a physical activity–related text message weekly. These were preprepared to be sent automatically each week and provided direct advice and a web link for further information. In addition, the telephone coaching program provided free, confidential health support to participants to reach personalized lifestyle goals concerning diet, physical activity, alcohol, and body weight [17]. The coaching was available in multiple languages through an interpreter service. The practice nurses conducted a 6-week health check in which they reviewed and revised the participants’ health goals. Additionally, general practitioners conducted a 12-week health review. Text messages reminded participants to attend these follow-up visits.

### Participants

Individuals were eligible for this study if they were aged 40-74 years, had a BMI of ≥28 and blood pressure levels recorded in the clinical software within the last 12 months, spoke English or Arabic, and had access to a smartphone or tablet. Potential participants were ineligible if they fulfilled any of the following exclusion criteria: recent weight loss (ie, >5% in the past 3 months), taking weight loss drugs (ie, orlistat or phentermine), diagnosed with insulin-dependent diabetes or cardiovascular disease (ie, angina, myocardial infarction, heart failure, heart valve disease, or stroke), cognitive impairment, or physical impairment disallowing them to perform moderate physical activity.

### *mysnapp* Design

The *mysnapp* content was based on a web-based platform designed to help individuals control and maintain their health data and information to manage their health [10]. Research by Webb et al [18] and DiFilippo et al [19] into behavior change through mobile and electronic platforms informed the app design, including goal setting and self-monitoring, and additional methods to interact with individuals, mainly text messaging. The *mysnapp* app consisted of 4 core modules that allowed users to (1) set physical activity– and diet-based goals, (2) monitor their progress over the past 6 weeks, (3) take notes in a diary, and (4) learn about healthy eating and physical activity. Users could choose from the following goal options: set daily servings of fruits or vegetables or physical activity minutes; aim to drink fewer soft drinks, eat smaller portions, or eat fewer snacks or takeaway foods. In the self-monitoring module, they entered how many days of the week they achieved their goals. The educational material consisted of short text summaries and fact sheets about healthy foods, portion sizes, discretionary beverage consumption, physical activity benefits in English or Arabic, and links to simple exercise videos on YouTube.

### Study Measures

#### App Use Measure

Data were collected on app use, specifically, when the study participants in the intervention group had an app account set up.

#### App Engagement Measures

Data were collected on the participants’ app use directly through *mysnapp*. Each month, a cumulative data report was created about app logins and interactions with the different app modules from each participant for 12 months. App engagement included active days, duration of app use, and frequency of accessing app modules.

#### Other Intervention Component Measures

The data from the 6-week health check with the practice nurses (ie, occurrence) and the telephone coaching (ie, occurrence and completion status) were the other intervention component measures.

#### Outcome Measures

The participants’ questionnaires at baseline and 6-month follow-up (ie, self-reported fruit and vegetable intake, and health literacy) were used.

Specifically, the diet questions were as follows: (1) How many servings of fruit do you usually eat each day? A serving is 1 medium-sized fruit such as an apple or 2 small-sized fruits or 1 cup of fruit pieces. (2) How many servings of vegetables do you usually eat each day? One serving is half a cup of cooked vegetables or 1 cup of salad vegetables. With the diet score being the portions of fruit intake (between 0 and a maximum of 2 per day) plus portions of vegetable intake (between 0 and a maximum of 5 per day) with a range of 0 to 7 based on the sum of fruit and vegetable scores. This diet measure has been validated against food frequency questionnaires [20].

Specifically, the Health Literacy Questionnaire domain 8 questions were used [11]: (1) find information about health problems; (2) find health information from several …. ; (3) get information about health so you…; (4) get health information in words you…; and (5) get health information by yourself. There is a 5-point response option scale for each question (cannot do or always difficult, usually difficult, sometimes difficult, usually easy or always easy). The scores are reported as averages for the domain (with a range between 1 and 5) with high scores representing higher health literacy.

Table 1 contains definitions for study measures. Duration of app use, active days, and consistent use had preset maximum values (365 days or 52 weeks); the values were capped when they exceeded the maximum.

**Table 1:** Measures and their definitions.

Measure		Type of variable	Explanation or definition
**App use measures**			
	App user	Binary and input variable	Study participants in the intervention group who had an app account set up
**App engagement measures**			
	Duration of app use	Continuous and input variable; maximum value: 365 days	Number of days between the first and last time a participant accessed the app
	Active days	Continuous and input variable; maximum value: 365 days	Number of days a participant accessed the app
	Consistent app use	Continuous and input variable; maximum value: 52 weeks	Number of consecutive weeks a participant accessed ≥1 time the app starting from the first app use
	App module use	Binary and input variable	Participant accessed ≥1 the corresponding app module (goal setting, progress tracking, diary, or education)
	Frequency of accessing app modules	Continuous and input variable	Number of times a participant accessed the corresponding app module (goal setting, progress tracking, diary, or education)
**Other intervention component measures**			
	Practice nurse-led health check	Categorical and input variable	Attended and not attended
	Telephone coaching	Categorical and input variable	Completed, not completed, and not participated
**Outcome measures**			
	Health literacy	Continuous and output variable	Health literacy, specifically the self-reported ability to find good quality health information, according to domain 8 of the Health Literacy Questionnaire [11], at baseline and 6-month follow-up. The scores were reported as averages for the domain (with a range between 1 and 5) with high scores representing higher health literacy.
	Diet score	Continuous and output variable	Self-reported daily fruit and vegetable intake at baseline and 6-month follow-up. Diet score was the portions of fruit intake (between 0 and a maximum of 2 per day) plus portions of vegetable intake (between 0 and a maximum of 5 per day) with a range between 0 and 7 based on the sum of fruit and vegetable scores.

### Data Analysis

Descriptive and inferential analyses in RStudio (with the programming language *R*; *R* Foundation for Statistical Computing) using a significance level of .05 for all statistical tests were conducted. Normally distributed continuous variables were summarized using the mean and SD, and nonnormally distributed continuous variables with median and IQR. Box plots compared continuous variables across the categories of nonnumerical variables [21]. Normality was tested using the Shapiro-Wilk normality test [22-24]. The 2-sided Welch *t* test was performed to compare the means of continuous variables between 2 subgroups (eg, participants using *mysnapp* versus those not using it) for normally distributed continuous variables [25]. Alternatively, the Wilcoxon signed rank test with continuity correction comparing the medians of nonnormally distributed continuous variables between 2 subgroups was used [26,27]. The Kruskal-Wallis rank-sum test was performed for more than 2 subgroups and nonnormally distributed continuous variables [28]. Pearson chi-square test with Yates continuity correction was used to test for associations between 2 categorical variables and the 2-sided Fisher exact test was used when there were less than 5 participants in any cell of the contingency table of expected frequencies [29-31].

For objective 4, we used 1-sided tests to assess whether app use versus nonapp use, or app engagement was associated with health literacy or diet between baseline and 6-month follow-up. Correlations between the app engagement and health literacy or diet score were measured with the Kendall rank correlation test (if variables did not follow a normal distribution) or Pearson product-moment correlation test (if they followed a normal distribution) [32,33].

## Results

### App Users

In total, 120 participants received the intervention, of which 62 (52%) people chose to use *mysnapp*. Among the 62 app users, 38 (61%) also opted for telephone coaching. Table 2 shows the results for the first objective, comparing the demographic characteristics of the participants who chose not to use *mysnapp* to those who decided to use it. There were no significant differences between app users and nonapp users.

**Table 2:** Demographic characteristics of participants in the intervention group (N=120).

Variables	Nonapp users (n=58)	App users (n=62)	Test statistics for differences between groups	*P* value
Age (years), mean (SD)	58 (8)	61 (9)	*t*_115_=–1.56	.12
Women, n (%)	28 (48)	32 (52)	*χ*^2^_1_<0.1	.86
Born in Australia, n (%)	27 (47)	39 (63)	*χ*^2^_1_=2.6	.11
Preferred language is English, n (%)	54 (93)	58 (94)	OR^a^ 0.93, 95% CI 0.16-5.26	>.99
Hospital admission in past 12 months, n (%)	15 (26)	12 (19)	*χ*^2^_1_=0.4	.53
Location New South Wales, n (%)	50 (86)	49 (79)	*χ*^2^_1_=0.6	.43

^a^OR: odds ratio.

### App Engagement

The median duration of app use was 52 (IQR 4-95) days. Further, 2 participants used *mysnapp* weekly throughout the 12 months (Table 3). Active days ranged from 1 to 117 days, with a median of 5 (IQR 2-10) days. The median number of weeks participants consistently used *mysnapp* from baseline was 1 (IQR 1-2). Of the 62 app users, 60 (97%) opened the goal setting module, 55 (89%) the education module, 39 (63%) the progress tracking module, and 25 (39%) the diary. Table 3 shows the consistency of app use and how many modules the app users accessed over the entire period of the intervention. Of the 19 app users who had opened 3 of the 4 modules, 17 (89%) had accessed the goal setting, progress tracking, and education modules. Among the 16 who had opened 2 modules, 14 (88%) had accessed the goal setting and education modules.

**Table 3:** Consistency of app use and frequency of accessing app modules (n=62).

Variables and values		Participants, n (%)	
**Consistent app use (weeks)**			
	1		45 (73)
	2-4		10 (16)
	5-19		5 (8)
	20-52		2 (3)
**Number of modules accessed**			
	0		1 (2)
	1		5 (8)
	2		16 (26)
	3		19 (31)
	4		21 (34)
**Frequency of accessing the goal setting module**			
	0-3		54 (87)
	4-7		6 (10)
	8-15		2 (3)
	>15		0 (0)
**Frequency of accessing the progress tracking module**			
	0-3		43 (69)
	4-7		11 (18)
	8-15		6 (10)
	>15		2 (3)
**Frequency of accessing the diary module**			
	0-3		48 (77)
	4-7		5 (8)
	8-15		4 (6)
	>15		5 (8)
**Frequency of accessing the education module**			
	0-3		41 (66)
	4-7		12 (19)
	8-15		6 (10)
	>15		3 (5)

### Association With Other Intervention Components

**Figure 1:**
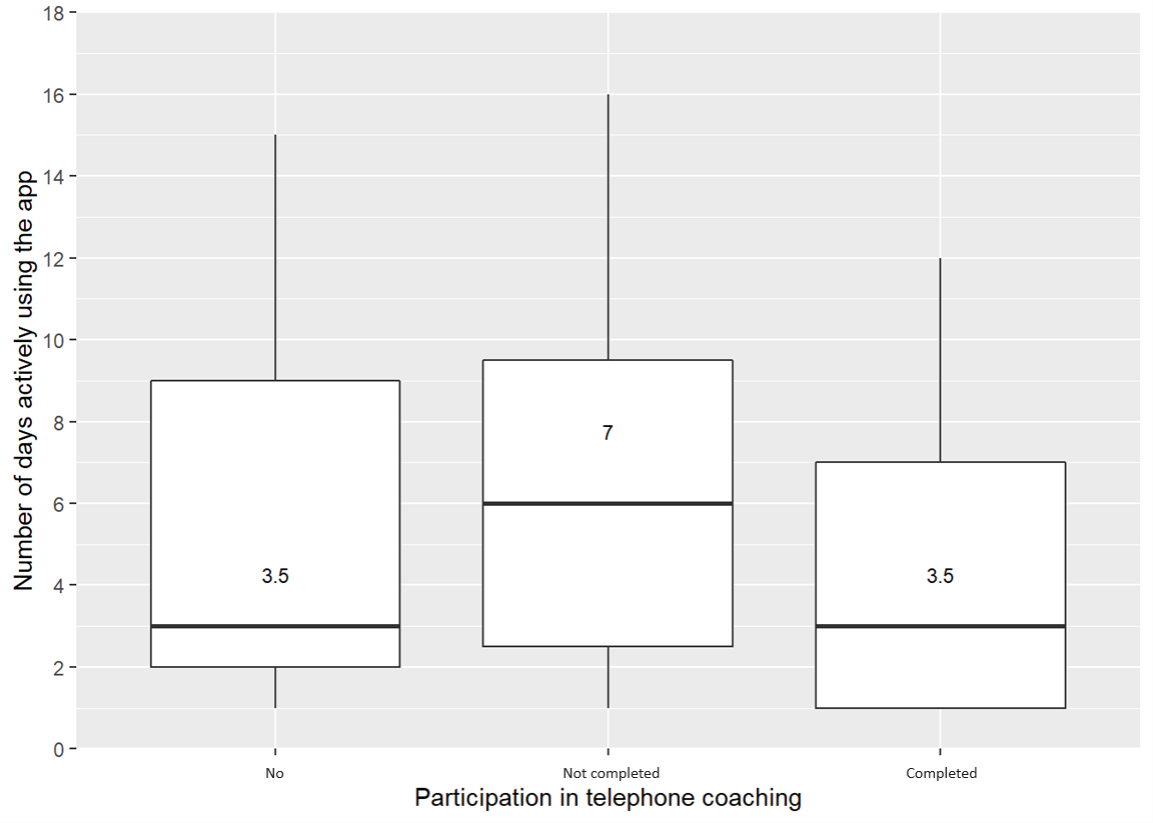
Box plots of the number of days actively using mysnapp depending on the participation in telephone coaching; outliers excluded (two for no telephone coaching: 30 and 105 days, one for not completed: 117 days, and one for completed: 105 days).

The difference in telephone coaching uptake between the app and nonapp users was statistically significant (Freeman-Halton extension of 2-sided Fisher exact test *P*<.001, Table 4). The median number of days using *mysnapp* for the app users who completed the telephone coaching was 3.5 (IQR 1-7) days, for the app users who did not complete the telephone coaching it was 7 (IQR 2.5-9.5) days, and for the app users who did not undertake the telephone coaching it was 3.5 (IQR 2-9) days (Figure 1). The difference in median active days by telephone coaching completion status was not statistically significant (*χ*^2^_19_=13.2, *P*=.83).

**Table 4:** Association of app use with other intervention components (N=120).

Other intervention components and status			Nonapp users (n =58), n (%)	App users (n=62), n (%)		Test for differences between groups	
**Telephone coaching program**							Freeman-Halton extension of Fisher exact test (2-tailed) *P*<.001
	Not participated	47 (81)		24 (39)			
	Not completed	8 (14)		16 (26)			
	Completed	3 (5)		22 (35)			
**A 6-week health check**							Fisher exact test (2-tailed) *P*=.007
	Not attended	54 (93)		46 (74)			
	Attended	4 (7)		16 (26)			

The difference in the attendance rate of the 6-week health check between app users and nonusers was significant (2-sided Fisher exact test *P*=.007, Table 4). Those app users who attended the 6-week health check with the practice nurse did not have significantly more active days using *mysnapp* (median active days for 6-week health check attendees: 6, IQR 2-10 days, and for nonattendees: 4, IQR 2-10 days; *W*=374, *P*=.46).

### Impact of App Use and App Engagement on Behavioral and Biomedical Outcome Measures

Differences in outcome measures between app users and nonusers, and app engagement were not significant (Tables 5 and 6) for study outcomes which were shown to be significantly improved at 6 months (ie, health literacy and diet).

**Table 5:** Health literacy and diet score at 2 time points for app and nonapp users, test for significant changes, and sensitivity analysis.

Outcome variable and measure		App users (n=62)			Nonapp users (n=58)			Test statistic^a^
		Baseline	6 months	Baseline		6 months		
**HLQ^b^ domain 8**								
	Data available, n (%)	52 (84)	44 (76)	50 (86)		20 (34)	N/A^c^	
	Median (IQR)	4 (4-5)	4 (4-5)	4 (4-4)		4 (4-5)	*W*=230.5, *P*=.10	
**Diet score**								
	Data available, n (%)	57 (92)	54 (93)	46 (74)		20 (34)	N/A	
	Mean (SD)	3 (2-5)	4 (4-5)	3 (2-4)		4 (3-5)	t_36_*=*0.32, *P*=.37	

^a^Test for greater change in app users versus nonapp users from baseline to 6 months.

^b^HLQ: Health Literacy Questionnaire.

^c^N/A: not applicable.

**Table 6:** Correlation between app engagement and change in health literacy or diet score.

Outcome variable and measure for app use		Test statistics for differences
**HLQ^a^ domain 8**		
	Active days	*z*=–0.24, *P*=.81, τ=–0.03
	Consistent app use	*z*=0.43, *P*=.67, τ=0.06
**Diet score**		
	Active days	*z*=0.55, *P*=.58, τ=0.07
	Consistent app use	*z*=0.43, *P*=.67, τ=0.06

^a^HLQ: Health Literacy Questionnaire.

## Discussion

### Principal Results

The overall aim of this study was to assess *mysnapp* app use within the HeLP-GP trial and its association with study outcomes shown to have significantly improved (health literacy and diet) at 6 months. With regard to the specific objectives, (1) there were no significant differences in demographics between participants who used *mysnapp* and those who did not; (2) among app users, the median duration of app use was 52 days, with a median of 5 active days; (3) more participants who chose to use *mysnapp* also attended the 6-week health check with the practice nurse and opted for telephone coaching; and (4) there was no association between app use and study outcomes shown to have significantly improved (health literacy and diet) at 6 months.

### Length and Frequency of App Use and Module Access

Turner-McGrievy et al [34] aimed to identify the best criteria for defining adherence to dietary self-monitoring with mobile devices when predicting weight loss. They found that adherence, defined as the number of days participants tracked at least 2 meal times, explained the most variance in weight loss at 6 months [34]. We were not able to measure this because the diary, available for recording meals, could also be used for other reasons such as activities, appointments, plans for the future, and thoughts about progress. In the study by Jacobs et al [35], they analyzed data from 7680 users of a commercial weight loss app; high adherence to self-monitoring (ie, logging at least 1 food event within a reasonable time after a meal) was associated with increased weight loss. However, they also found that app users with higher adherence rates had significantly lower body weight at baseline than those with lower adherence rates [35]. The analysis only comprised people who entered data in the app at least once a week for 12 weeks. In our study, 4.9% (n=3) of the app users were still entering data at week 12. Analyzing data from the same commercial app, Carey et al [36] found significant differences in 7 different engagement measures (ie, number of articles read, meals logged, steps recorded, messages to coach, exercise logged, weigh-ins, and days with 1 meal logged per week) between app users with moderate or high weight loss (ie, 5%-10% or >10% body weight loss, respectively) and individuals with no change in body weight (ie, ±1% body weight). Their analysis indicated that people with moderate to high weight loss engaged with all app sections [36]. In our study, only 34% (n=21) of the app users had accessed all of the modules.

### Impact of App Use and App Engagement on Behavioral and Biomedical Outcome Measures

Other studies showed promising results for weight loss apps, for example, Carter et al [37], Patel et al [5], and Antoun et al [38]. Specifically, Carter et al [37] conducted a pilot study of 128 volunteers who are overweight comparing a smartphone app (My Meal Mate) with a website and paper diary. They found the mean weight loss over 6 months for the app was higher (4.6 kg, 95% CI 3.0-6.2) than for the diary group (2.9 kg, 95% CI 1.1-4.7) or the website group (1.3 kg, 95% CI 0.1-2.7). Antoun et al [38] in their review of 34 studies that evaluated the use of smartphones for weight loss found an overall mean loss of 2.8 kg (95% CI 2.6-3.0) at 6 months. Patel et al [5] found that consistent tracking was associated with greater weight loss than inconsistent tracking at 6 months (2.1 kg, 95% CI 0.3-4.0). A difference between these studies and ours was that they did not specifically target disadvantaged populations with low health literacy. Therefore, their apps were more complex than ours. In contrast, Lanpher et al [39] developed a weight loss intervention suitable for individuals with low health literacy. A computer algorithm automatically allocated the self-monitoring goals (eg, no sugary drinks, no snacking after dinner, eating 5 fruits and vegetables a week). Participants reported whether they achieved the goals via interactive voice response calls [39]. The algorithm decided which goals to assign next based on previous adherence to goals so that individuals would rather receive goals to which they were receptive [40]. They also received tailored skills training through verbal calls and materials, one-on-one counseling calls, and a membership at the gym [39]. The results showed that the intervention group maintained or lost weight over 12 months, independent of their level of health literacy [38].

Bennett et al [40] extended the intervention to comprise a mobile app. They evaluated its effectiveness in a randomized controlled trial including socioeconomically disadvantaged patients with increased cardiovascular risk by comparing the intervention to usual care [40]. The app used interactive voice responses or text messaging to simplify self-monitoring, like in the previous study. Additionally, participants received in-person coaching and personalized feedback messages immediately after entering data [40]. The intervention group achieved meaningful weight loss, with more than 40% of participants reducing their body weight by at least 5% compared to 17% of participants in the usual care group [39]. Comparing this intervention to ours raises the question of whether the way people had to select and track their goals in our app contributed to the low engagement and the nonsignificant findings. Locke and Latham [41] explained that goal commitment, goal importance, self-efficacy, feedback, and task complexity act as moderators between goals and performance. Potentially, the app did not sufficiently address all 5 moderators.

### Association With Other Intervention Components

This study showed that *mysnapp* users were more likely to attend the 6-week face-to-face health check with the practice nurse and to participate in the telephone coaching program than nonusers. Potentially, these individuals were more motivated to lose weight and, therefore, more willing to engage in the other intervention components. Another explanation could be that participants who opted for several intervention components felt more committed to study participation and, therefore, made more use of the individual intervention components. Griauzde et al [42] proposed a similar hypothesis in their mobile health–based prediabetes intervention study; they assumed that participants who received a more robust intervention were more committed to the study and subsequently more likely to complete the 12-week survey. Hutchesson et al [43] concluded that adding nondigital components, such as face-to-face visits and telephone coaching, to mobile health interventions can improve participants’ accountability even though these additional features may not be necessary for the intervention’s effectiveness.

### Limitations

The plan for the randomized controlled trial was to recruit 800 study participants; however, only 215 individuals were able to be recruited (120 in the intervention and 95 in the control group) [13]. Further, despite targeting low socioeconomic areas, this study failed to recruit many participants with low health literacy. One needs to be cautious when interpreting the results of this study due to the small sample size and the high dropout. Despite considerable efforts and additional time to recruit participating practices and patients, the anticipated sample size was not achieved. Research by Perkins et al [44] has shown an ongoing issue with recruitment through Australian general practices. Another problem with the study was that the uptake of intervention components was determined by the clinician and patient. Thus, some chose to just have the app and others to just have the phone coaching. Additionally, the study may not be generalizable to other settings. Since recruitment was from 2 Australian urban areas, results could differ in rural areas or other urban areas. Diet score and health literacy level were self-reported, posing a risk of bias. Further, caution is required when interpreting the results in the context of low health literacy because the baseline health literacy levels were higher than anticipated [12]. According to data from the National Health Survey 2018, the health literacy level in this study’s sample was comparable to that of Australians who are overweight or those with obesity in the general population [45]. A potential explanation is that this study’s requirements (randomization, completing the questionnaire, and undertaking the health check) stopped people with low health literacy from participating. This rationale is in line with results from Kripalani et al [46], who found that people with low health literacy or numeracy were significantly less interested in participating in research.

### Conclusions

There was no association between app use and study outcomes shown to have significantly improved (health literacy and diet) at 6 months. Recruitment and engagement were difficult for this study in disadvantaged populations with low health literacy. A potential explanation could be related to the self-selection of the goals and the weekly submission of the goal achievements. The practice nurses assisted participants at the beginning with the selection of goals. However, these may not have been relevant to participants, and nurses did not receive specific training in selecting meaningful goals for individuals.

However, app users were more likely to attend the 6-week health check and participate in telephone coaching, suggesting that participants who opted for several intervention components felt more committed to this study.

## Appendix

Multimedia Appendix 1 CONSORT-eHEALTH checklist (V 1.6.1).

## Abbreviations

GP: general practice
HeLP-GP: Health eLiteracy for Prevention in General Practice
