# Correction: Mobile App Intervention of a Randomized Controlled Trial for Patients With Obesity and Those Who Are Overweight in General Practice: User Engagement Analysis Quantitative Study

**DOI:** 10.2196/58507

**Published:** 2024-04-02

**Authors:** Vera Helen Buss, Margo Barr, Sharon M Parker, Alamgir Kabir, Annie Y S Lau, Siaw-Teng Liaw, Nigel Stocks, Mark F Harris

**Affiliations:** 1 Centre for Primary Health Care and Equity University of New South Wales Sydney Australia; 2 Australian Institute of Health Innovation Macquarie University Sydney Australia; 3 School of Population Health University of New South Wales Sydney Australia; 4 Adelaide Medical School University of Adelaide Adelaide Australia

In “Mobile App Intervention of a Randomized Controlled Trial for Patients With Obesity and Those Who Are Overweight in General Practice: User Engagement Analysis Quantitative Study” (JMIR Mhealth Uhealth 2024;12:e45942), the authors noted two errors. The following corrections have been made:

Under “Methods”, the third paragraph of the “Outcome Measures” subsection has been changed from:

Specifically, the Health Literacy Questionnaire domain 8 questions were the following [11]: please indicate how difficult or easy the following tasks are for you now: (1) find information about health problems; (2) find health information from several different places; (3) get information about health so you are up to date with the best information; (4) get health information in words you understand; and (5) get health information by yourself. There is a 5-point response option scale for each question (cannot do or always difficult, usually difficult, sometimes difficult, usually easy or always easy). The scores are reported as averages for the domain (with a range between 1 and 5) with high scores representing higher health literacy.

And now reads as follows:

The Health Literacy Questionnaire domain 8 questions were used [11]:(1) find information about health problems; (2) find health information from several …. ; (3) get information about health so you…; (4) get health information in words you…; and (5) get health information by yourself. There is a 5-point response option scale for each question (cannot do or always difficult, usually difficult, sometimes difficult, usually easy or always easy). The scores are reported as averages for the domain (with a range between 1 and 5) with high scores representing higher health literacy.

Additionally, in Table 1 the definition of the “Health literary” row has been changed from:

Health literacy, specifically the self-reported ability to find good quality health information, according to domain 8 of the Health Literacy Questionnaire [9], at baseline and 6-month follow-up.

To reference a different source, this now appears as:

Health literacy, specifically the self-reported ability to find good quality health information, according to domain 8 of the Health Literacy Questionnaire [11], at baseline and 6-month follow-up.

The correction will appear in the online version of the paper on the JMIR Publications website on April 2, 2024, together with the publication of this correction notice. Because this was made after submission to PubMed, PubMed Central, and other full-text repositories, the corrected article has also been resubmitted to those repositories.

